# A Role for Both Conformational Selection and Induced Fit in Ligand Binding by the LAO Protein

**DOI:** 10.1371/journal.pcbi.1002054

**Published:** 2011-05-26

**Authors:** Daniel-Adriano Silva, Gregory R. Bowman, Alejandro Sosa-Peinado, Xuhui Huang

**Affiliations:** 1 Department of Chemistry, The Hong Kong University of Science and Technology, Clear Water Bay, Kowloon, Hong Kong; 2 Department of Biochemistry, Medicine School, Universidad Nacional Autónoma de México, México D.F., México; 3 Department of Chemistry, Stanford University, Stanford, California, United States of America; National Cancer Institute, United States of America and Tel Aviv University, Israel

## Abstract

Molecular recognition is determined by the structure and dynamics of both a protein and its ligand, but it is difficult to directly assess the role of each of these players. In this study, we use Markov State Models (MSMs) built from atomistic simulations to elucidate the mechanism by which the Lysine-, Arginine-, Ornithine-binding (LAO) protein binds to its ligand. We show that our model can predict the bound state, binding free energy, and association rate with reasonable accuracy and then use the model to dissect the binding mechanism. In the past, this binding event has often been assumed to occur via an induced fit mechanism because the protein's binding site is completely closed in the bound state, making it impossible for the ligand to enter the binding site after the protein has adopted the closed conformation. More complex mechanisms have also been hypothesized, but these have remained controversial. Here, we are able to directly observe roles for both the conformational selection and induced fit mechanisms in LAO binding. First, the LAO protein tends to form a partially closed encounter complex via conformational selection (that is, the apo protein can sample this state), though the induced fit mechanism can also play a role here. Then, interactions with the ligand can induce a transition to the bound state. Based on these results, we propose that MSMs built from atomistic simulations may be a powerful way of dissecting ligand-binding mechanisms and may eventually facilitate a deeper understanding of allostery as well as the prediction of new protein-ligand interactions, an important step in drug discovery.

## Author Summary

Protein-ligand interactions are crucial to chemistry, biology and medicine. Many studies have been conducted to probe the mechanism of protein-ligand binding, leading to the development of the induced fit and conformational selection models. Unfortunately, experimentally probing the atomistic details of protein-ligand binding mechanisms is challenging. Computer simulations have the potential to provide a detailed picture of molecular recognition events. In this study, we construct kinetic network models from atomistic simulations to elucidate the mechanism by which the LAO protein binds to its ligand. Because the LAO protein completely encompasses its substrate in the bound state, it has generally been assumed that it operates via an induced fit mechanism. We find that both the conformational selection and induced fit mechanisms play important roles in LAO binding. Furthermore, we have identified a number of parallel pathways for binding, all of which pass through a single gatekeeper state, which we refer to as the encounter complex state because the protein is partially closed and only weakly interacting with its substrate.

## Introduction

Molecular recognition plays important roles in many biological processes. For example, enzymes must recognize their substrates and drugs must be designed to have specific binding partners. Unfortunately, our understanding of how ligand binding occurs remains incomplete. In particular, the role that protein dynamics play in protein-ligand binding is unclear.

Two popular models for protein-ligand binding are the induced fit and conformational selection mechanisms. Both attempt to explain how a protein could transition from an unbound conformation to a bound conformation in complex with a ligand. In the induced fit model—introduced by Koshland [1]—the ligand first binds to the protein in its unbound conformation and this binding event induces the protein to transition to the bound state. Such models have been applied to many protein-protein and protein-DNA/RNA binding systems [2], [3], [4]. The conformational selection (or population shift) model [5], [6], [7], [8], [9], [10], [11], [12] is a popular alternative to the induced fit mechanism. In this model, the intrinsic dynamics of the protein lead it to constantly transition between a stable unbound conformation and a less stable bound conformation. The ligand can then bind directly to the bound conformation, thereby stabilizing the bound state and increasing its population relative to the unbound state. The conformational selection model has recently gained popularity in antibody or small ligand binding systems [10], . Some docking studies have also tried to exploit conformational selection by generating an ensemble of protein structures and docking small molecules against each of them in the hopes of identifying a transiently populated bound conformation that will be stabilized by the ligand [13].

Many recent studies have attempted to determine whether a variety of systems under different conditions can be best described by the induced fit or conformational selection model [14], [15], [16], [17], [18], [19], [20], [21]. For example, Okazaki *et. al.* [20] have found that strong and long range protein-ligand interactions favor the induced fit model, while weak and short range interactions favor the conformational selection model. Based on an analytic model, Zhou has suggested that the determining factor in ligand binding is the timescale for transitioning between the unbound and bound states with and without the ligand [18]. He found that conformational selection dominates when transitioning between the unbound and bound states is slow, while the induced fit mechanism dominates when this transition is fast. Many studies have proposed that conformational selection and induced fit are not mutually exclusive; instead, a blend of these two models may best describe most realistic systems [15], [17], [18], [20], [22], [23]. For example, Zagrovic and coworkers [17] have suggested that conformational selection and induced fit play equal roles in ubiquitin binding based on their analysis of NMR structures. However, in many cases, it is still difficult to dissect the chemical details of binding mechanisms. While it is clear that the bound and unbound states of a protein and their respective interactions with a ligand molecule are of great importance [15], [18], [20], [21], it may also be important to take other conformational states into account. Protein dynamics are ultimately determined by their underlying free energy landscapes, whose ruggedness frequently gives rise to numerous metastable regions-sets of rapidly mixing conformations that tend to persist for extended periods of time.

**Figure 1:**
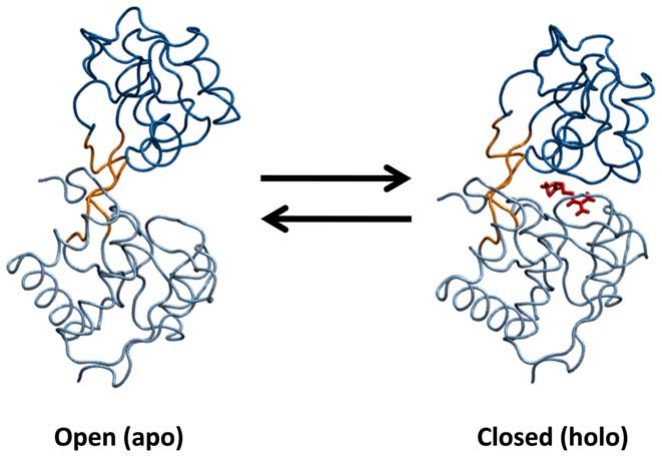
The open (PDB ID: 2LAO) and closed (PDB ID: 1LAF) states of the Lysine-, Arginine-, Ornithine-binding (LAO) Protein. The ligand, Arginine, is shown in red.

In this work, we use Markov state models (MSMs) to map out the relevant conformational states in LAO binding and describe mechanistic details of this process. MSMs are a kinetic network model and a powerful approach to automatically identifying metastable states and calculating their equilibrium thermodynamics and kinetics [24], [25], [26], [27]. MSMs focus on metastable regions of phase space, while there also exist other kinetic network models to study transition state [28]. MSMs partition conformational space into a number of metastable states; such that intra-state transitions are fast but inter-state transitions are slow. This separation of timescales ensures an MSM is Markovian (i.e. that the probability of transitioning from state *i* to state *j* depends only on the identity of *i* and not any previously visited state) and allows MSMs built from short simulations to model long timescale events. Many recent studies have demonstrated how MSMs can provide insight into drastic conformational changes like protein and RNA folding [26], [29], [30], [31], [32]. Here we demonstrate that MSMs built with a hierarchical clustering algorithm [30] can capture the mechanism by which the Lysine-, Arginine-, Ornithine-binding (LAO) protein, one of Periplasmic Binding Proteins (PBPs), binds to arginine. The LAO protein has a high binding affinity and undergoes large-scale domain rearrangements from an open to a closed state upon ligand binding [33], [34], [35], [36] (see Fig. 1), making it a valuable model system for probing the coupling between protein conformational changes and binding.

Many have assumed that PBP binding occurs via the induced fit mechanism because the ligand is completely encapsulated by the protein in the bound state (see Fig. S1). Experimental studies of many PBPs support the induced fit mechanism, where the closure of the domains is triggered by the binding of the ligand [33], [34], [36], [37], [38], [39], [40]. However, a few experimental studies indicate that some PBPs (including GGBP [41] and ChoX [42]) are able to reach the closed conformation in the absence of the ligand. This has been suggested as a sign of the conformational selection mechanism [38]. Furthermore, recent NMR studies with paramagnetic relaxation enhancement (PRE) of maltose-binding protein (MBP) identified a minor (∼5%) un-liganded partially closed form. This partially closed state is in equilibrium with the open state and, therefore, is available for the binding of the ligand, which may further facilitate the transition to the bound state. This work suggests a more complex binding mechanism where both conformational selection and induced fit play significant roles [43], but since the ligand was not present during the experiments, it is unclear exactly what roles the two mechanisms may play.

With our MSM, we can directly monitor the mechanism of LAO binding and assess the role of both conformational selection and induced fit. Our model suggests that three dominant states need to be considered to adequately describe LAO binding and that both conformational selection and induced fit play important roles in the transitions between these states. The third dominant state in our model—besides the open and closed states—is only partially closed and weakly bound to the ligand; therefore, we refer to it as the encounter complex state. The ligand can induce the protein to transition from the open state to the encounter complex; however, the ligand-free protein can also transition to the encounter complex state, indicating an important role for conformational selection. In contrast, on our dataset the ligand-free protein never sampled the closed state, this suggest that that the closed state in the absence of the ligand may represent a very high free energy state and that once the ligand reaches the binding site an induced fit mechanism is responsible for transitions from the encounter complex to the closed state.

## Results

### Model validation by *ab initio* prediction of the bound state and binding kinetics

Before drawing system-specific conclusions from a simulation study, it is important to first test the model against existing experimental data. MSMs built using the Super-level-set Hierarchical Clustering (SHC) algorithm [30] greatly facilitate this task by decomposing a system's conformational space into its constituent metastable regions and describing the thermodynamics and kinetics of each. For instance, one can easily extract representative conformations from each state, determine the equilibrium probability of each state, or calculate the rates of transitioning between sets of states and compare to experimental results. In this study, we describe protein conformations by the opening and twisting angles between their two domains [44] and the location of the ligand because these degrees of freedom describe the slowest dynamics of the system (see Fig. S2). We then construct a 54-state MSM using SHC (See Methods for details of MSM construction). The dominant conformational states in our model are displayed in the Fig. S3.

**Figure 2:**
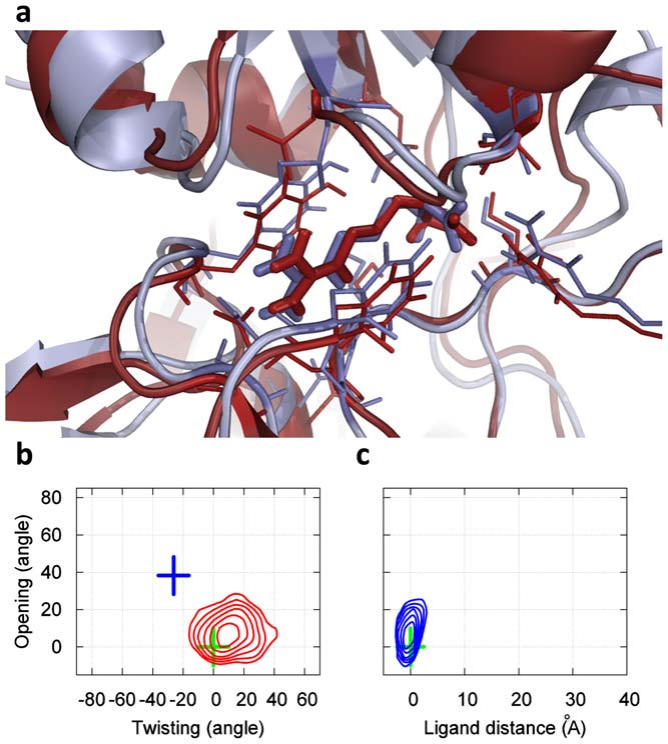
The bound state of our MSM for LAO binding (which is also the most populated state, having an equilibrium population of 74.9%). **(a)** A snapshot from our simulations (red) achieves a 1.2 Å RMSD to the X-ray bound state (blue, PDB ID: 1LAF). The RMSD is computed from the protein Cα atoms that are within 8 Å to the center of mass of the ligand in the X-ray bound state (Residues 9–15, 17–19, 30, 50–53, 55–56, 67–74, 77, 83, 88, 90–92, 117–124, 141–143, 159–162, 164, 190–191 and 194–196). If all-protein Cα atoms are included the RMSD is 1.8 Å. **(b)** Free energy plot of the protein opening angle versus twisting angle. The bin size is (5°, 5°), and the interval between two adjacent contour levels is 0.5 KT. The green and blue crosses correspond to X-ray structures of the bound and apo conformations respectively. (c) Free energy plot of the opening angle versus the distance between the ligand and the binding site. The bin size is (1.5 Å, 5°), and interval between contour levels is 0.5 KT.

Fig. 2 demonstrates that our model is capable of *ab initio* prediction of the bound state. As described in the Methods section, no knowledge of the bound state was included at any stage of our simulations or model construction. Based on the high binding affinity measured in experiments (K_d_ ∼14 nM) [45] we postulated that the bound state should be the most populated state in our model. Indeed, representative conformations from our most populated state (having an equilibrium population of 74.9%) agree well with the crystal structure of the bound state, with an RMSD to the crystal structure of the binding site as little as 1.2 Å, as shown in Fig. 2A. Moreover, Figs. 2B and 2C show that the crystal structure of the bound state lies within the minimum of the most populated free energy basin. These figures also show that our model's bound state covers a relatively large region of phase space, suggesting that it is flexible, possibly to accommodate favorable interactions with all four of LAOs binding partners (L-lysine, L-arginine, L-ornithine and L-histidine). The structural properties of the remaining states are also consistent with experiments (see the Text S1 for more details). For example, many of the unbound states also contain partially closed protein conformations, consistent with NMR experiments on another PBP protein: MBP [43].

Our model is also in reasonable agreement with the experimentally measured binding free energy and association rates. For example, from the MFPT from all unbound states to the bound state, our model predicts an association timescale of 0.258 ± 0.045 µs (see Methods for calculation details). Since rates are proportional to the exponential of the free energy barrier, an 8-fold difference in rates roughly corresponds to a 2 kT difference in the height of the free energy barrier. Therefore, our result is in reasonable agreement with the experimental value of ∼2.0 µs found in the highly homologous HisJ protein [46] (see Methods for similarity between LAO and HisJ protein). We also estimate a binding free energy of −8.46 kcal/mol using the algorithm introduced by van Gunsteren and co-workers [47], which is also in reasonable agreement with the experimental value of −9.95 kcal/mol for the LAO protein (see Methods for calculation details). Together, this agreement between theory and experiment suggests that our model is a sufficiently good reflection of reality to make hypotheses about details of the binding mechanism.

Arriving at these conclusions with a single long simulation would have been quite difficult due to the slow timescales involved. For example, transitioning from the bound state to an unbound state takes 2.15±0.51 µs on average. Therefore, observing enough transitions to gather statistics on the binding and unbinding rates in a single simulation would require that it be tens of µs long. Such simulations are now possible [31], [48] but are still challenging to perform. Moreover, scaling the long simulation approach to millisecond timescales is still infeasible. MSMs built from many µs timescale simulations, however, have already proven capable of capturing events in a 10 millisecond timescale [49] and can likely scale to even slower processes.

### Insights into the mechanism of LAO binding

**Figure 3:**
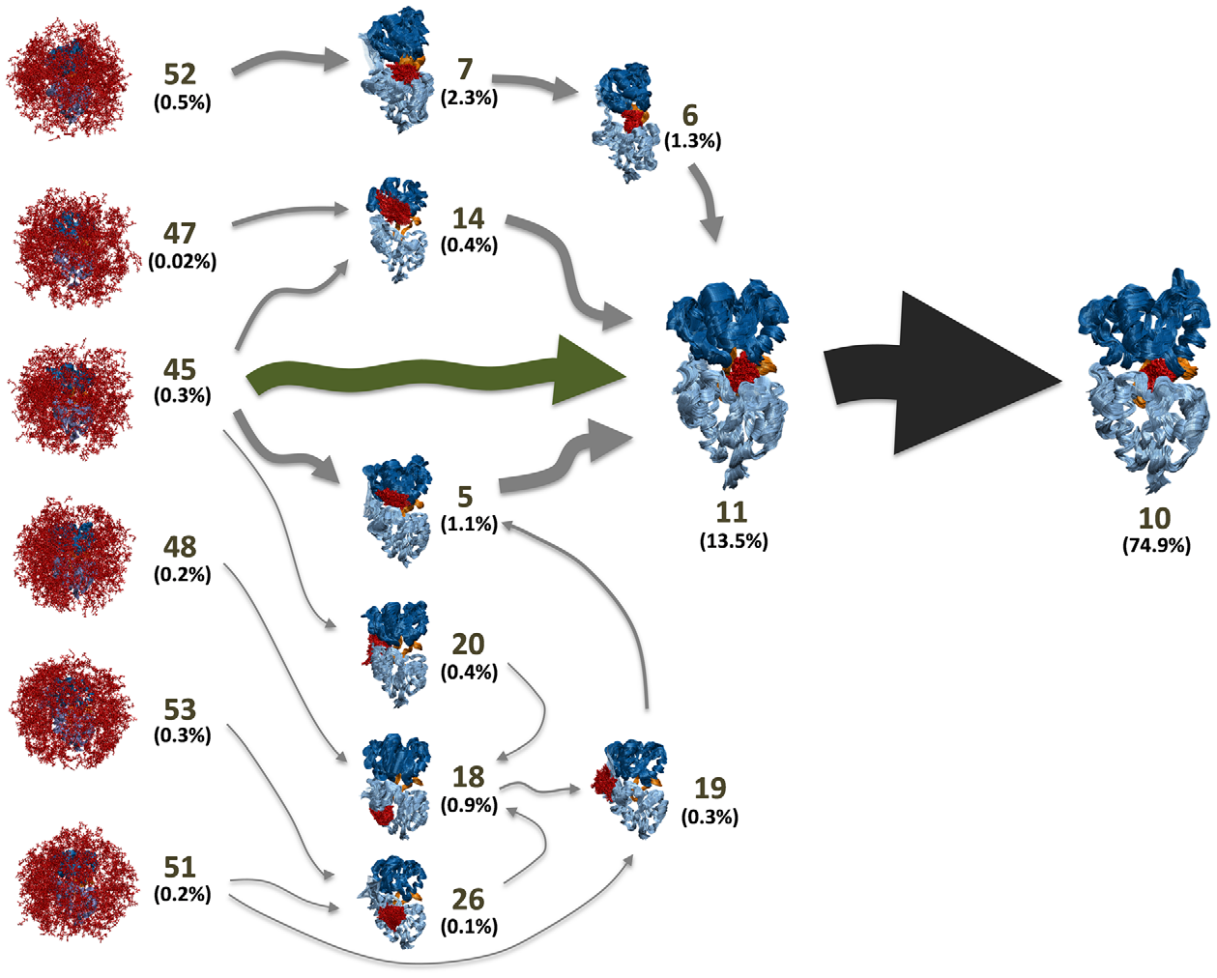
Superposition of the 10 highest flux pathways from the unbound states to the bound state. The flux was calculated using a greedy backtracking algorithm [31], [66] applied to a 54-state MSM generated with the SHC algorithm [30]. These pathways account for 35% of the total flux from unbound states to the bound state. The arrow sizes are proportional to the interstate flux. State numbers and their equilibrium population calculated from MSM are also shown. The conformational selection and induced pathways from the unbound states to the encounter complex state is shown in green and grey arrows respectively.

**Figure 4:**
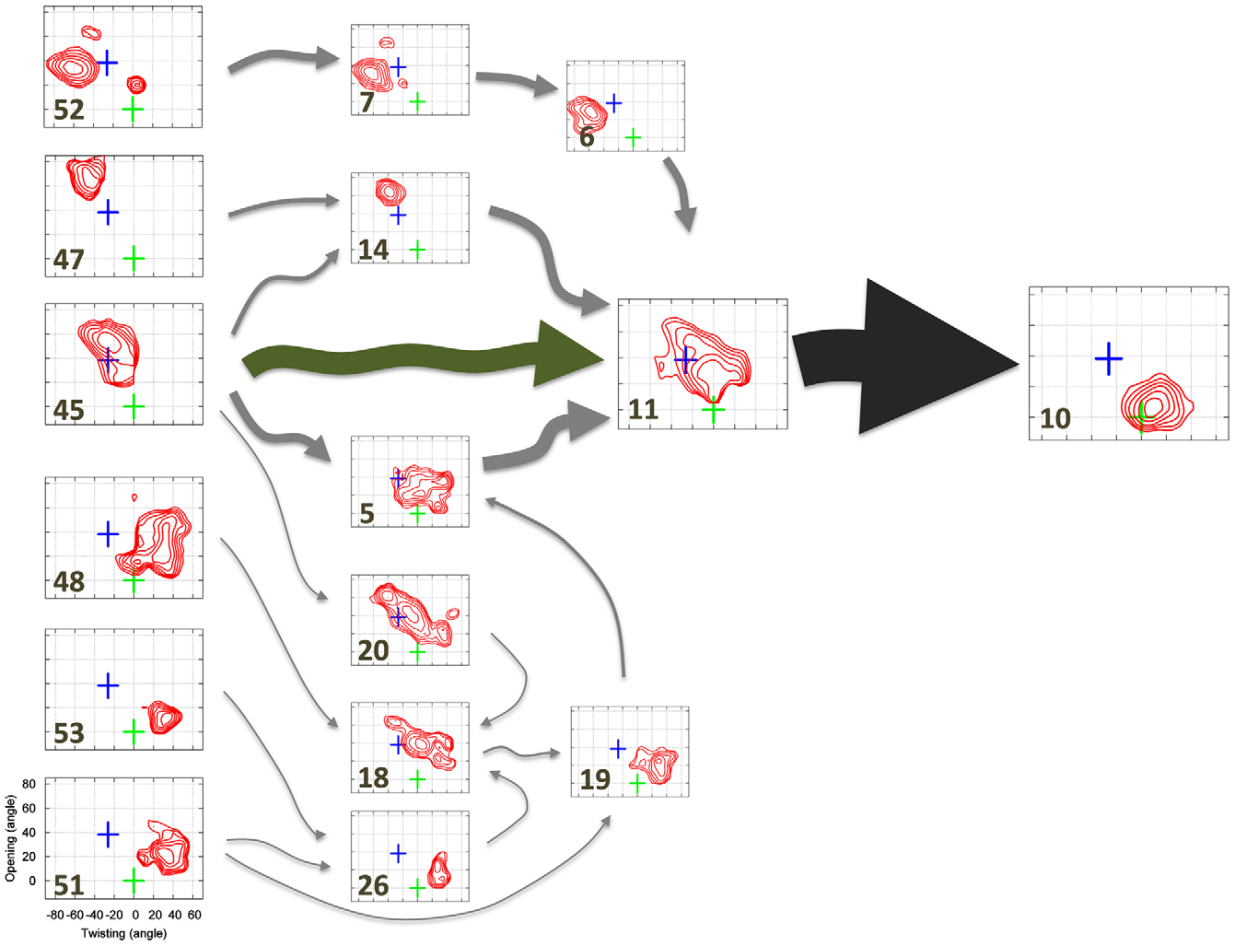
Superposition of the 10 highest flux pathways from the unbound states to the bound state as in Fig. 2A but with representative structures replaced with free energy plots of the protein opening angle versus twisting angle. The green and blue crosses correspond to X-ray structures of the bound and apo conformations respectively. The bin size is (5°, 5°), and the interval between contour levels is 0.5 KT (same as Fig. 2b). The conformational selection and induced pathways from the unbound states to the encounter complex state is shown in green and grey arrows respectively.

**Figure 5:**
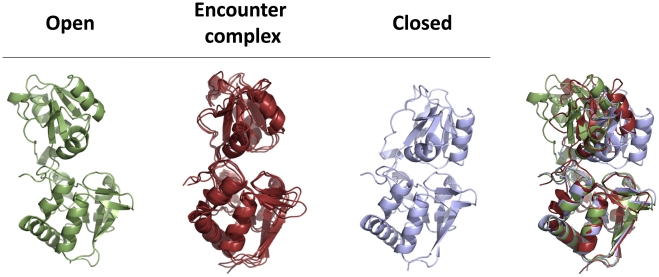
Structural comparisons between encounter complex and X-ray apo (or open) and bound (or closed) structures for the LAO protein. The X-ray apo (PDB ID: 2LAO) and bound structure (PDB ID: 1LAF) are shown in green and light blue respectively. Three representative conformations from the encounter complex state are superimposed and shown in red. These three conformations are representative of 10,000 randomly selected conformations from the encounter complex state (i.e. they have the smallest protein Cα RMSD to all the rest of the randomly selected conformations and are, therefore, the most central/typical of the state). In the right panel, the open, closed X-ray structures are overlaid with one of the representative conformations from the encounter complex state.

In addition to predicting experimental parameters, MSMs are also useful for mapping out conformational transitions like protein-ligand binding. For example, Figs. 3 and 4 show the 10 highest flux pathways from any of the unbound states in our model to the bound state. All ten pathways pass through an obligatory, gatekeeper state (state 11) that we refer to as the encounter complex state because the protein is partially closed and only weakly interacting with the ligand (see Figs. 3, 4 and State 11 in Fig. S3). In the encounter complex (see Fig. 5) the two lobes of the LAO protein are structurally very similar to those in both the apo and bound X-ray structures (with RMSD less than 2 Å, see Table S1). Therefore the conformational change between crystal structures and the encounter complex could be achieved through a rigid body rotation. We also found that in the encounter complex the ligand was stacked between the lobe I Tyr14 and Phe52 and protrudes upward to interact with the lobe II Thr121. These contacts are also observed in the X-ray bound structure. (see Fig. S4). To further support our conclusion that the encounter complex state is an obligatory step in ligand binding, we have calculated that the average timescale for transitioning from the unbound states to the encounter complex state is 0.190±0.037 while the average timescale for transitioning from the unbound states to the bound state is 0.258 ± 0.045 µs. The average timescale for transitioning from the encounter complex state to the bound state is 0.090±0.015 µs (see Methods for calculation details). Thus, the unbound protein will typically transition to the encounter complex before reaching the bound state.

The top ten paths from the unbound states to the encounter complex can be divided into two sets, one that is best described by conformational selection and one that is better described by the induced fit mechanism. For example, the pathway from state 45 directly to 11 operates through conformational selection (see green arrow in Fig. 4): in the unbound state 45 the protein and ligand are not interacting but the protein conformations are very similar to those in the encounter complex. Since the protein adopts similar conformations in these two states, the ligand can always bind to a pre-existing encounter-complex-like (state 11 like) protein conformation (the conformational selection mechanism). The binding kinetics of this conformational selection pathway is quite rapid, having a mean first passage time for transitioning from the unbound state 45 to the encounter complex state 11 of 0.220±0.054 µs, and this pathway accounts for ∼45% of the flux of the top ten pathways from unbound states to the bound state.

The second group of pathways to the encounter complex, which together account for ∼55% of the flux may be better described by the induced fit mechanism. In general, these pathways start off in conformations that are much more open or twisted than the encounter complex. Next, the system transitions to one or more intermediate states where the ligand is interacting with the protein at (or near) its binding site, though the protein is still quite open or twisted. Finally, the protein-ligand interactions induce a transition to the encounter complex state. For example, the pathway starting from state 47, passing through state 14, and ending at state 11 falls into this category (see Fig. 4).

**Figure 6:**
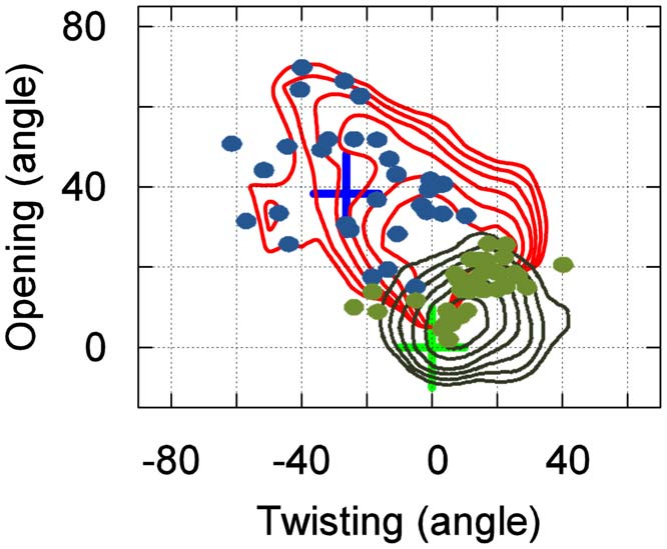
Overlay of free energy plots of the protein opening angle versus twisting angle for the encounter complex (red) and bound state (blue). Green dots correspond to where transitions from the encounter complex to the bound state occur. Blue dots correspond to where transitions into the encounter complex from other states occur. Only transitions without re-crossing are counted (minimum residence time in the final state after the transition is 6 ns).

Transitions from the encounter complex to the bound state are best described by the induced fit mechanism. When the system enters the encounter complex state, the protein is generally in a relatively open conformation (opening angle within 20° to 70°, see Fig. 6). However, when the system leaves the encounter complex state to enter the bound state, the protein is mostly in a more closed conformation (opening angle smaller than 30°, see Fig. 6). Thus, it appears interactions with the ligand induce the protein to close. Furthermore, our model predicts that the encounter complex-to-bound transition (0.090±0.015 µs) is much faster than the encounter complex-to-unbound transition (1.927±0.499 µs), so the encounter complex is not likely to diffuse back to the unbound state instead of converting into the bound state. In addition, the protein never samples fully-closed conformations in the absence of the ligand in our simulations. Together, these observations indicate that the induced fit mechanism should dominate transitions from the encounter complex to the bound state.

### Mis-bound states

We have also identified a number of metastable mis-bound states, like states 4 and 20 in Fig. S3. In these states, the ligand interacts with the protein outside of the binding site. For example, in state 20 (population ∼0.4%) the ligand is bound to the hinge region between the two domains of the protein. Transitioning from a mis-bound state to the bound state generally requires passing through an unbound state (see Fig. S5). Therefore, these states are mostly off pathway and likely slow down the overall binding kinetics.

## Discussion

The LAO protein is a member of the PBP family, which is responsible for transporting low molecular weight ligands from the outer to the inner membrane in the ABC transport mechanism of Gram-negative bacteria [34], [50]. Crystal structures for this system have shown that the binding site is completely closed-off in the bound state [33], [37], making it impossible for the ligand to enter the binding site after the protein has adopted the closed conformation (see Fig. S1). Therefore, it has often been proposed that LAO binding occurs through an induced fit mechanism [51], [52], [53].

Our observations of LAO binding indicate that, like protein folding, ligand binding is a multi-state process with parallel pathways. All top 10 pathways pass through a gate-keeper state that we refer to as the encounter complex state because the protein is partially closed and only weakly interacting with the ligand. The system can reach this state through either the induced fit mechanism or conformational selection. Rather than being a transient state, this encounter complex is quite metastable. Once in the encounter complex state, the ligand is able to quickly induce protein conformational changes that lead to a transition to the fully closed, bound state.

Other systems may operate through a similar mechanism such as other PBPs. Indeed, our model is consistent with Tang *et. al.* [43]'s findings for MBP. Specifically, they discovered a partially closed state in equilibrium with the open state for the apo protein. Thus, this state is available for the binding of the ligand to form the encounter complex through the conformational selection mechanism. Next, the binding could facilitate the transition to the bound state via the induced fit mechanism. Their model was proposed mainly based on NMR experiments in the absence of the ligand, but our simulations directly observed this interplay at atomic resolution. Furthermore, our results suggest that transitions from the open to the partially closed state occur via a combination of conformational selection and induced fit mechanisms. For several PBPs including MBP and HisJ, the rate constant measured by the stopped flow experiments is proportional to the ligand concentration, indicating a simple two-state binding mechanism [46]. However, as discussed by Tang *et. al.* [43], these stopped flow measurements may not be able to capture the intermediate encounter complex state because the overall binding timescale is extremely rapid, e.g. a few hundred nanoseconds for LAO.

More broadly, there may also exist other proteins with closed active sites that have metastable encounter complex states. Sullivan *et. al.* [16] suggested that in such encounter complex state substrate-enzyme interactions are almost identical to the active state, while the enzyme has not yet reached the active form. Furthermore, the enzyme must operate by an induced fit mechanism to reach the active form because of the closure of the enzyme would prevent the substrate from entering the active site. This model is consistent with our findings for the LAO protein. However, in order to reach this encounter complex state, we suggest that both induced fit and conformational selection may play important roles. In general, other proteins with closed active sites may also make use of both conformational selection and induced fit to reach the encounter complex. However, the relative contributions of these mechanisms may vary depending on factors like the relative strength of the protein-ligand interactions [20].

The ability to map out the details of LAO binding using MSMs is an important step towards a deeper understanding of protein-ligand interactions for this system. Future application of these methodologies to other systems could even lead to the identification of general principles of protein-ligand interactions and allostery. This knowledge may also greatly aid in computational drug design. For example, it may not always be possible to identify all the relevant states via other structural methods, like crystallography. Using MSMs, however, one can hope to identify the most important relevant states and design small-molecules to specifically stabilize one or more states over the others. Future work with improved force fields and greater sampling could also greatly enhance our understanding of protein-ligand interactions. However, we stress that the present work lays out the methodology that would be employed in such future research.

### Conclusions

**Figure 7:**
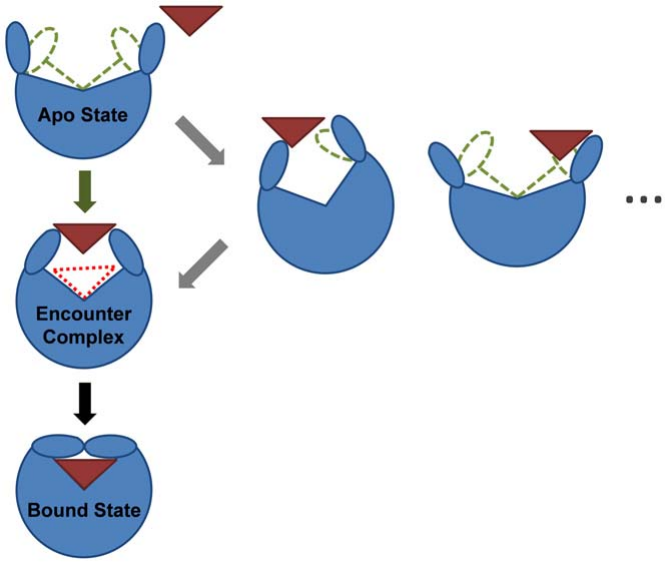
A schematic diagram describing the proposed two-step binding mechanism for proteins in steric occlusion of the direct binding of the ligands. The first step is the transition from the apo to the encounter complex state. In this step, multiple pathways exist where both conformational selection and the induced fit mechanisms play important roles. The second step is the transition from the encounter complex to the bound state, where the induced fit mechanism is adopted.

In this study, we demonstrate the power of MSMs for understanding protein-ligand interactions using the LAO protein as a model system. Our results indicate that LAO binding is a two-step process involving many states and parallel pathways. In the first step, the ligand binds to a partially closed protein to form an encounter complex. Both the conformational selection and induced fit mechanisms play significant roles in this step. In the second step, the system transits from the encounter complex state to the bound state via the induced fit mechanism. This two-step binding mechanism (see Fig. 7 for schematic diagram of the binding mechanism) may also be used by other systems, such as other PBP proteins, enzymes with closed active sites, and systems where the apo protein dynamics rarely visits the bound conformation. Future applications of MSMs with improved force fields, greater sampling, and to other protein-ligand interactions will reveal how general this mechanism is, aid in understanding allostery, and lay a foundation for improved drug design.

## Methods

### Simulation dataset

We have performed 65 molecular dynamics simulations, each 200 ns long, of the LAO protein from the organism *Salmonella typhimurium* and one of its ligands, L-arginine. Ten simulations were started from the open protein conformation (PDB ID: 2LAO [37]) with the ligand at more than 25 Å away from the binding site. The other simulations were initialized from conformations randomly selected from the first ten simulations. We saved conformations every 20ps with a total of more than 650,000 conformations. Among these MD simulations, we have observed multiple binding events and unbinding events (see Fig. S6 and Text S2). The protein was solvated in a water box with 11,500 SPC waters [54] and 1 Na^+^ ion. All the simulations were performed using the GROMACS 4.0.5 simulation package [55] with the GROMOS96 force field [56]. The simulation system was minimized using a steepest descent algorithm, followed by a 250 ps MD simulation applying a position restraint potential to the protein heavy atoms. The simulations were performed under isothermal-isobaric conditions (NPT) with P = 1 bar and T = 318 K, using Berendsen thermostat and Berendsen barostat with coupling constants of 0.1 ps^−1^ and 1 ps^−1^ respectively [57]. A cutoff of 10 Å was used for both VDW and short-range electrostatic interactions. Long-range electrostatic interactions were treated with the Particle-Mesh Ewald (PME) method [58]. Nonbonded pair-lists were updated every 10 steps. Waters were constrained using the SETTLE algorithm [59] and all protein bonds were constrained using the LINCS algorithm [60]. Hydrogen atoms were treated as virtual interaction sites, enabling us to use an integration step size of 5 fs [61].

### Markov State Model (MSM) state decomposition

We used MSMBuilder [27] and SHC [30] to construct the state decomposition for our MSM for LAO binding.

We first used the k-centers algorithm in MSMBuilder [27] to cluster our data into a large number of microstates. The objective of this clustering was to group together conformations that are so geometrically similar that one can reasonably assume (and later verify) that they are also kinetically similar.

Because we had to account for both the protein and ligand, we performed two independent clusterings; one based on the opening and twisting angle of the protein and one based on the relative position of the ligand (see Fig. S2). We then combined the two clusterings by treating them as independent sets. For example, M protein-based clusters and N ligand-based clusters would lead to a total of M×N clusters.

For the protein-based clustering, we created 50 clusters using the Euclidean distance between a vector containing the protein opening and twisting angles. The opening angle (see Fig. S2a) was defined as the angle between the normal vectors of the two planes defined by the center of masses of the following groups of C_α_ atoms:

- Plane-A: Residues 6–88 & 195–227; 162–168; and 121–127;
- Plane B: Residues 92–185; 162–168; and 121–127.

The twisting angle (see Fig. S2b) is the angle between the following two planes:

- Plane-A: Residues 6–88 & 195–227; 83–88 &194–199; and 92–97 & 156–161;
- Plane-B: Residues 92–185; 83–88 &194–199; and 92–97 & 156–161.

The strong correlation between opening and twisting angles of the protein and the two slowest eigenvectors from Principle Component Analysis (PCA) analysis of a 20 ns MD simulation started from the apo structure in the absence of the ligand demonstrates that they are a reasonable descriptor of the protein's conformation (see Figs. S2c and S2d). As a reference point, we note that the holo X-ray structure (PDB ID: 1LAF) [33] has both opening and twisting angles equal zero, while the apo X-ray structure (PDB ID: 2LAO) [37] has opening angle = 38.2° and twisting angle = −26.2°.

For the ligand-based clustering, we created 5000 clusters using the Euclidean distance between all heavy-atoms.

We then had to modify our clustering to account for the fact that the ligand dynamics fall into two different regimes (see Fig. S7): one where the ligand moves slowly due to interactions with the protein and one where the ligand is freely diffusing in solution. The existing clusters are adequate for describing the first regime. However, when the ligand is freely diffusing (more than 5 Å from the protein), the procedure outlined above results in a large number of clusters with poor statistics (less than ten transitions to other states). Better sampling of these states would be a waste of computational resources as there are analytical theories for diffusing molecules and a detailed MSM would provide little new insight. Instead, we chose to re-cluster these states using the same protein coordinates and the Euclidean distance between the ligand's center of mass (as opposed to the Euclidean distance between all ligand heavy-atoms). For this stage, we created 10 new protein clusters and 100 new ligand clusters.

After dropping empty clusters, this procedure yielded 3,730 microstates. Of these, 3,290 microstates came from the initial high resolution clustering and 440 came from the data that was reclustered at low resolution. To verify that the final microstate model is valid (Markovian) we plotted the implied timescales and found that they level off at a lag time between 2 and 6 ns (see Text S3 and Fig. S8a), implying that the model is Markovian for lag times in this range. Therefore, we can conclude that the microstates are sufficiently small to guarantee that conformations in the same state are kinetically similar.

We then lumped kinetically related microstates into macrostates using the SHC algorithm [30]. This is a powerful lumping method that efficiently generates more humanly comprehensible macrostate models (i.e. ones with fewer small macrostates arising from statistical error) than the PCCA algorithm currently implemented in MSMBuilder.

In SHC, one performs spectral clustering hierarchically using super level sets (or density levels) starting from the highest density level, thereby guaranteeing that highly populated meta-stable regions are identified before less populated ones. For SHC, we selected density levels L_high_ = [0.50, 0.55, 0.60, 0.65, 0.70, 0.75, 0.80, 0.85, 0.90, 0.95, 0.99] and L_low_ = [0.4, 0.95], for the high and low-density regions respectively. The low and high resolution states were lumped separately because the states in each set have different sizes, so it is difficult to compare their densities. We then combined these two sets of macrostates to construct an MSM with 54 macrostates. Once again, we used the implied timescales test to verify that the model is Markovian and found that a 6 ns lag time yields Markovian behavior (see Fig. S8b).

### Calculating transition matrices

To calculate a transition matrix using the above state decomposition we first counted the number of transitions between each pair of states at some observation interval (the lag time) to generate a transition count matrix, where the entry in row x and column y gives the number of transitions observed from sate x to state y. In particular, we use a sliding window of the lag time on each 200 ns trajectory with a 20 ps interval between stored conformations (i.e. each trajectory contains 10,000 conformations) to count the transitions. Because we use a hard cutoff between states, simulations at the tops of barriers between states can quickly oscillate from one state to the other, leading to an over-estimate of the transition rate between states [62]. To mitigate the effect of these recrossing events, we only counted transitions from state *x* to state *y* if the protein remained in state *y* for at least 300 ps before transitioning to a new state. To generate the transition probability matrix (where the entry in row *x* and column *y* gives the probability of transitioning from state *x* to state *y* in one lag time), we normalized each row of the transition count matrix.

### Mean First Passage Time (MFPT) calculation

We followed the procedure in Ref [63] to compute the mean first passage time **(MFPT)** from initial state ***i*** to final state ***f***, i.e. the average time taken to get from state ***i*** to state ***f***. In particular, the **MFPT (** ***X_if_*** **)** given that a transition from state *i* to *j* was made first is the time it took to get from state ***i*** to ***j*** plus the **MFPT** from state *j* to *f.* **MFPT (** ***X_if_*** **)** can be defined as,
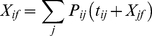
(1)where ***t_ij_*** **= 6 ns** is the lag time of the transition matrix **T**. The boundary condition is:
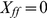
(2)


A set of linear equations defined by Equation **(1** **)** and **(2)** can be solved to obtain the **MFPT** ***X_if_***. We used bootstrapping to put error bars on MFPTs. That is, one-hundred new data sets were created by randomly choosing trajectories 130 times with replacement. We then calculated the MFPTs for each data set and reported their means and standard deviations. In MFPT calculations, the encounter complex was considered to contain state 11 and state 5, because state 5 also has features of the encounter-complex, though it plays a significantly smaller role (refer to the Text S1 for details).

### Calculation of the binding free energy and association rate

To compute the binding free energy ΔG from our simulations, we use the method introduced by van Gunsteren and co-workers [47]: 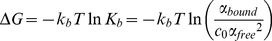
(3)where α_bound_ and α_free_ are the fractions of bound and free species respectively. c_0_ is the overall concentration of ligand. In our simulations, 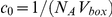, where N_A_ is Avogadro's number, and V_box_ is the volume of the simulation box. In our system, c_0_ = 0.0049 mol/L, T = 318K. We consider all the unbound states as free species, so that α_free_ = 1.75%_._ Thus, the bound species α_bound_ = 1−α_free_ = 98.25%, which contains all the states where the protein and ligand are in close contact. From Eq. (3), we have:
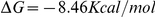



We can also derive the association rate. Given a system in equilibrium described by the protein ***P***, ligand ***L*** and the protein-ligand complex ***P•L***, the protein-ligand binding reaction can be written as:
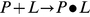
(4)


Rate equation can be written as
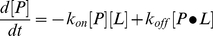
(5)Where k_on_ and k_off_ are forward and backward rate constants respectively. Since the forward reaction (i.e. association) only depends on k_on_, the rate equation for the forward reaction can be written as:
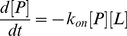
(6)The total concentrations of protein and ligand in the system are constant
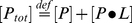
(7)

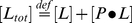
(8)Thus only one concentration among [P], [L], and [P•L] is independent. If we choose [P] as the independent concentration and then rate equation for forward reaction can be rewritten as:
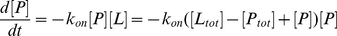
(9)If we consider the condition 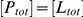, which is the case for our simulations:
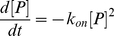
(10)We can solve Eq (10) with the initial condition 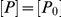 at t = 0:
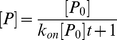
(11)We define the association timescale (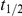) as the time when half of the protein (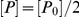) has associated with the ligand:
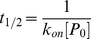
(12)


Since there is no experimental k_on_ rate constant available for the LAO protein, we choose for comparison the k_on_ from the Histidine binding (HisJ) protein. The LAO and HisJ proteins have considerable similarity both in structure and function. For example, both proteins are the same size (238 a.a.) and have a 70% sequence identity. In fact, if conservative mutations are taken into account the sequence identity increases to 83% [64]. These homologous proteins also bind to the same membrane receptor (HisQ/HisM/HisP) [37] and the same ligands (cationic L-amino acids) [65]. The RMSD between the X-ray crystal structures of holo LAO and HisJ bound to histidine (1LAG and 1HBP) is also quite small (Cα RMSD as low as 0.62 Å). The binding affinities of these proteins to their ligands are also similar (all about a nanomolar, though the binding affinities are not exactly the same [Histidine binds to HisJ most strongly, but binds to LAO most loosely]). The similarity between LAO and HisJ has also been discussed in detail in a previous study by Oh *et. al.* [65]. Therefore, we think it is a reasonable assumption that the LAO and HisJ proteins have similar binding kinetics.

For the Histidine binding protein, 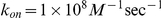. In our simulation, the initial concentration of protein is 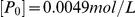, thus, the association timescale: 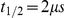



The experimental association timescale is about eight times slower than that computed from our simulations. However, we note that the only available experimental k_on_ was measured at 293K [46], while our simulations were performed at a higher temperature (318K) with faster kinetics. Thus, the difference between the experimental and simulation rates will be smaller at the same temperature. For the binding free energy, the experimental measurement was at an even lower temperature (277K) [45]. Thus, the experimental binding free energy at the temperature we simulated should be closer to our calculated value. 

## Supporting Information

Figure S1 The X-ray bound structure of LAO (PDB ID: 1LAF) from four different viewing angles shows that the protein completely encloses the ligand. Thus, unbound ligands cannot enter the binding site when the protein is closed.
(TIF)

Figure S2 (a) Opening and (b) twisting angles used to describe the motion of the protein. (c). The projection of conformations on the second eigenvector from Principle Component Analysis (PCA), and protein opening dihedral angle (see SI Sec 2 for detailed definition) as a function of time are shown in red and black respectively. The 20 ns simulation is started from protein in the closed state (PDB ID: 1LAF), but ligand was not included in the simulation. (d) Same as (c) except that the projection of conformations on the first eigenvector from PCA and protein twisting dihedral angle are plotted. In this system, the twisting and opening angles are correlated well with the first and second eigenvectors from PCA.
(TIF)

Figure S3 Populations of top 23 most populated macrostates (macrostate-population>0.1%) of the 54-state MSM. 1,000 conformations randomly selected from each macrostate are overlaid and shown from four different viewing angles. Free energy plots of the protein opening angle versus twisting angle (O, T) and the distance between the ligand and the binding site versus the opening angle (L,O) are displayed in red and blue respectively. The bin size is 5° for “O” and “T” and 1.5 Å for the ligand distance. The interval between two adjacent contour levels is 0.5 KT. The green and blue crosses correspond to the X-ray bound and apo structures respectively. For the free energy plots the x and y axis-scales are the same than those found in the Fig. 2 of the main text.
(TIF)

Figure S4 The three representative conformations from the encounter complex state based on RMSD of the (a) protein C-α atoms and (b) binding site plus the ligand (heavy atoms of the residues within a cutoff of 8 Å from the ligand in the crystal structure with PDB ID: 1LAF) are compared to (c) the crystal bound structure of LAO (PDB ID: 1LAF). The protein is shown in cartoon representation while the residues within a cutoff of 8 Å from the ligand in the crystal structure of 1LAF are shown in lines. In these conformations, we found the ligand side chain interacts with the lobe I Tyr14 and Phe52 through cation-Pi interactions. From five out of the six conformations, we also observed that the NH_3_COO^−^ group of the ligand protrudes upward to interact with the lobe II residue Thr121. The same interactions are also observed in the X-ray bound structure.
(TIF)

Figure S5 Superposition of the 5 highest flux pathways from mis-bound states to the bound state. The flux was calculated using a greedy backtracking algorithm from a 53-state Markov State Model (MSM) generated with the SHC algorithm. The arrow size is proportional to the interstate flux. (a) Representative structures and equilibrium populations are shown for each state. (b) Free energy plots of protein opening versus twisting angle are shown. The macrostate number is inserted in each of the free energy plots.
(TIF)

Figure S6 The ligand distance to the binding site vs. simulation time was plotted for 12 MD simulations where binding (11 simulations) or unbinding (1 simulation, gray shaded) events were observed.
(TIF)

Figure S7 Ligand rotational autocorrelation functions for the unbound states (blue), the encounter complex state (green), and the bound state (red) are shown in the left panel. Autocorrelation functions generated from different trajectories are overlaid in the same figure. On the right panel, a schematic figure illustrates that the ligand rotates quickly when it is far away from the protein but the ligand rotation is restrained when it interacts with the protein. Thus, when constructing MSMs, we only consider the ligand center of mass motion when the ligand does not have strong interactions with the protein but we consider motion of all the ligand heavy atoms when the ligand is strongly interacting with the protein.
(TIF)

Figure S8 Twenty slowest implied timescales as a function of lag time computed from (a) MSM containing 3730 microstates and (b) MSM containing 54 macrostates. Both plots level off at a lag time of ∼4 ns. Thus we choose 6 ns to construct final MSMs.
(TIF)

Table S1 Averaged RMSD of the LAO protein Lobes I and II between three representative encounter complex conformations and the apo and bound X-ray structures. Cα atoms of the Lobe I residues 6-88 & 195-227 or Lobe II residues 92-185 were included in the RMSD calculations. The structural alignment and RMSD calculation were performed separately for each Lobe.
(DOC)

Text S1 Structural features of the macrostates.
(PDF)

Text S2 Binding/unbinding transitions observed in the MD dataset.
(PDF)

Text S3 Implied timescales calculation.
(PDF)
