# Supplementary figures and images for: A Role for Both Conformational Selection and Induced Fit in Ligand Binding by the LAO Protein
Source: PLoS Comput Biol. 2011 May 26;7(5):e1002054. doi: 10.1371/journal.pcbi.1002054 (PMC3102756; doi:10.1371/journal.pcbi.1002054)

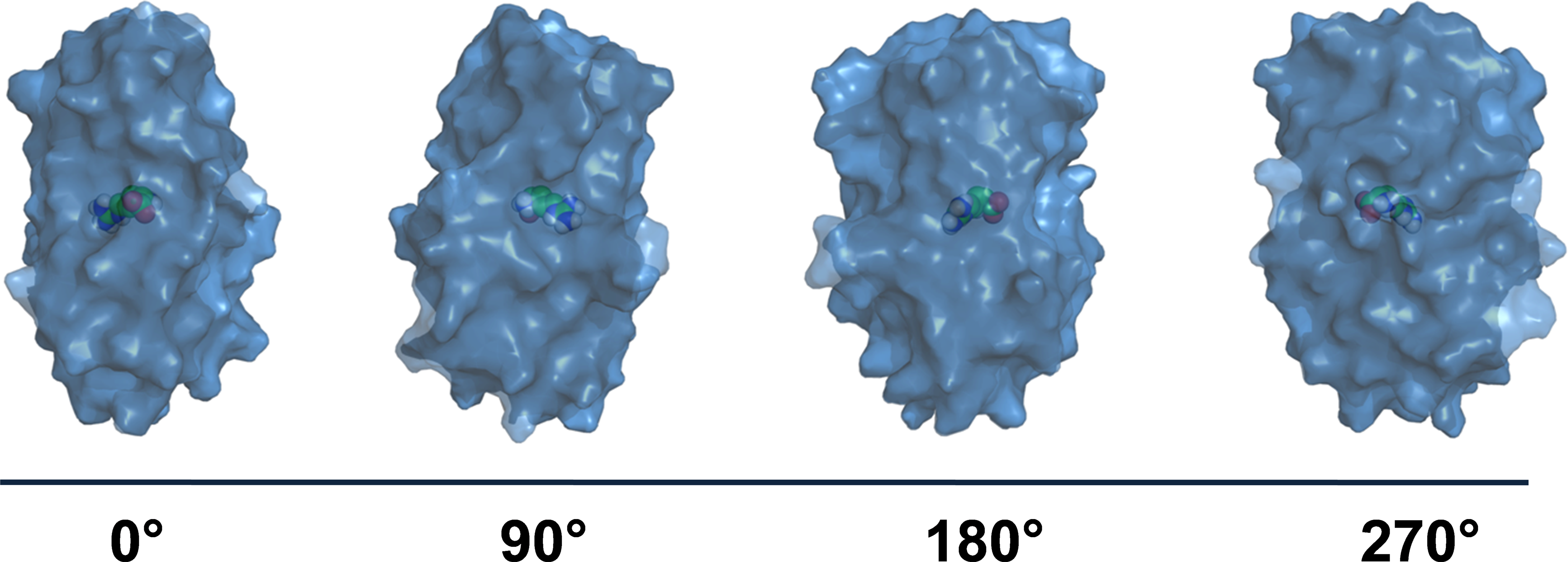

Supplement: Figure S1 — The X-ray bound structure of LAO (PDB ID: 1LAF) from four different viewing angles shows that the protein completely encloses the ligand. Thus, unbound ligands cannot enter the binding site when the protein is closed. (TIF) [file pcbi.1002054.s001.tif]

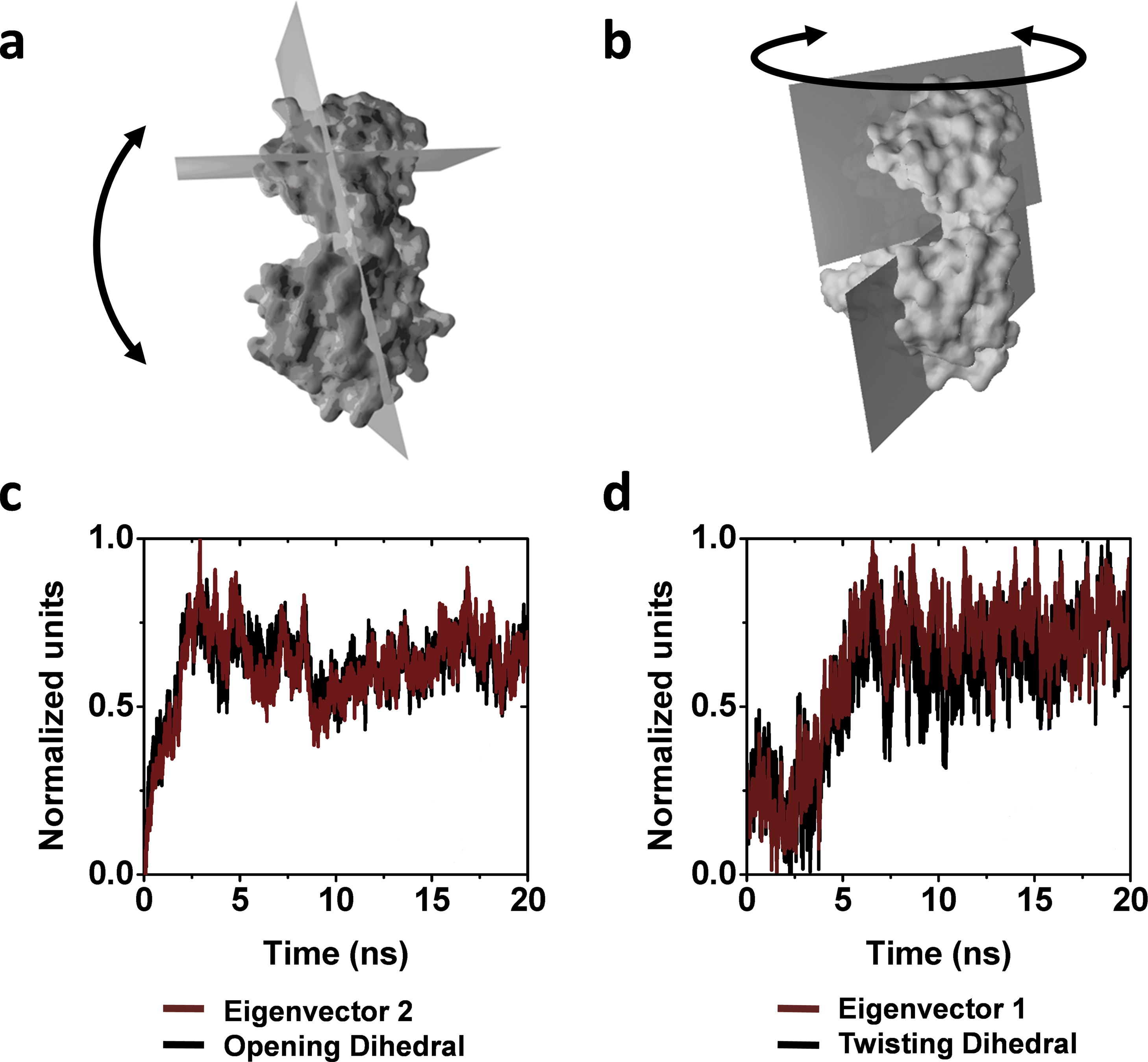

Supplement: Figure S2 — (a) Opening and (b) twisting angles used to describe the motion of the protein. (c). The projection of conformations on the second eigenvector from Principle Component Analysis (PCA), and protein opening dihedral angle (see SI Sec 2 for detailed definition) as a function of time are shown in red and black respectively. The 20 ns simulation is started from protein in the closed state (PDB ID: 1LAF), but ligand was not included in the simulation. (d) Same as (c) except that the projection of conformations on the first eigenvector from PCA and protein twisting dihedral angle are plotted. In this system, the twisting and opening angles are correlated well with the first and second eigenvectors from PCA. (TIF) [file pcbi.1002054.s002.tif]

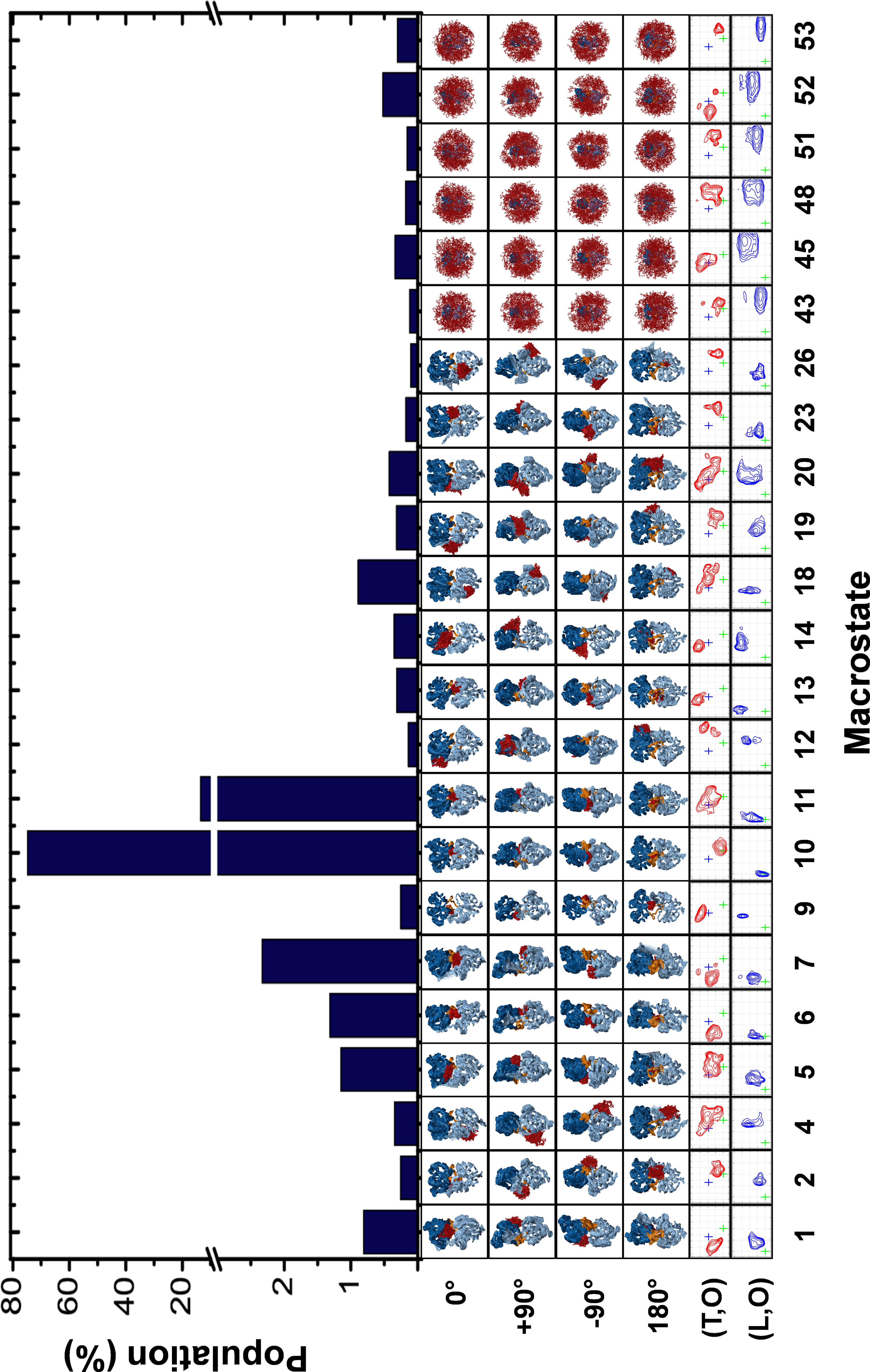

Supplement: Figure S3 — Populations of top 23 most populated macrostates (macrostate-population>0.1%) of the 54-state MSM. 1,000 conformations randomly selected from each macrostate are overlaid and shown from four different viewing angles. Free energy plots of the protein opening angle versus twisting angle (O, T) and the distance between the ligand and the binding site versus the opening angle (L,O) are displayed in red and blue respectively. The bin size is 5° for “O” and “T” and 1.5 Å for the ligand distance. The interval between two adjacent contour levels is 0.5 KT. The green and blue crosses correspond to the X-ray bound and apo structures respectively. For the free energy plots the x and y axis-scales are the same than those found in the Fig. 2 of the main text. (TIF) [file pcbi.1002054.s003.tif]

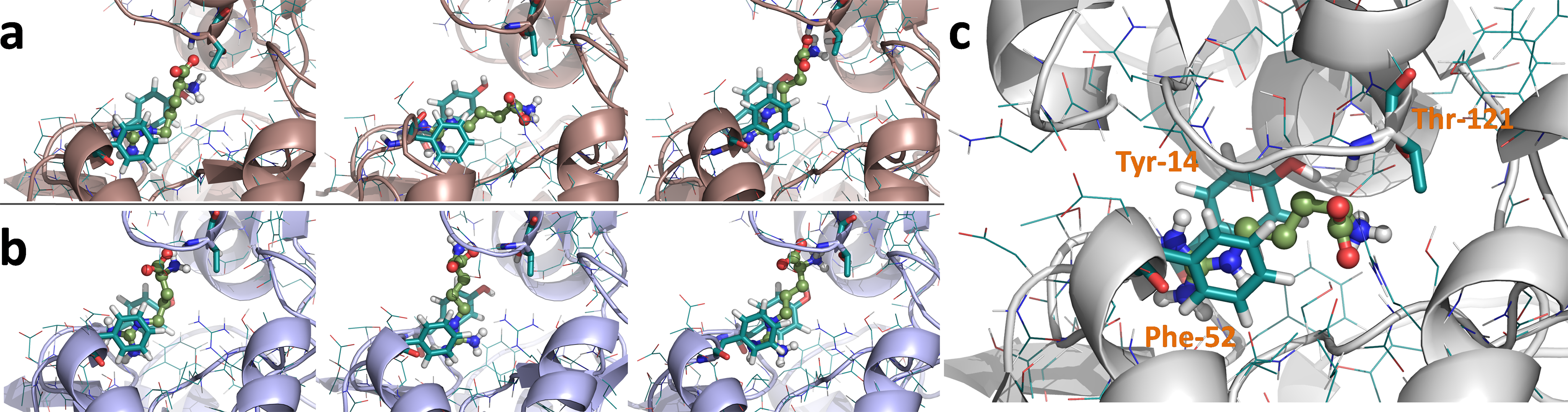

Supplement: Figure S4 — The three representative conformations from the encounter complex state based on RMSD of the (a) protein C-α atoms and (b) binding site plus the ligand (heavy atoms of the residues within a cutoff of 8 Å from the ligand in the crystal structure with PDB ID: 1LAF) are compared to (c) the crystal bound structure of LAO (PDB ID: 1LAF). The protein is shown in cartoon representation while the residues within a cutoff of 8 Å from the ligand in the crystal structure of 1LAF are shown in lines. In these conformations, we found the ligand side chain interacts with the lobe I Tyr14 and Phe52 through cation-Pi interactions. From five out of the six conformations, we also observed that the NH3COO− group of the ligand protrudes upward to interact with the lobe II residue Thr121. The same interactions are also observed in the X-ray bound structure. (TIF) [file pcbi.1002054.s004.tif]

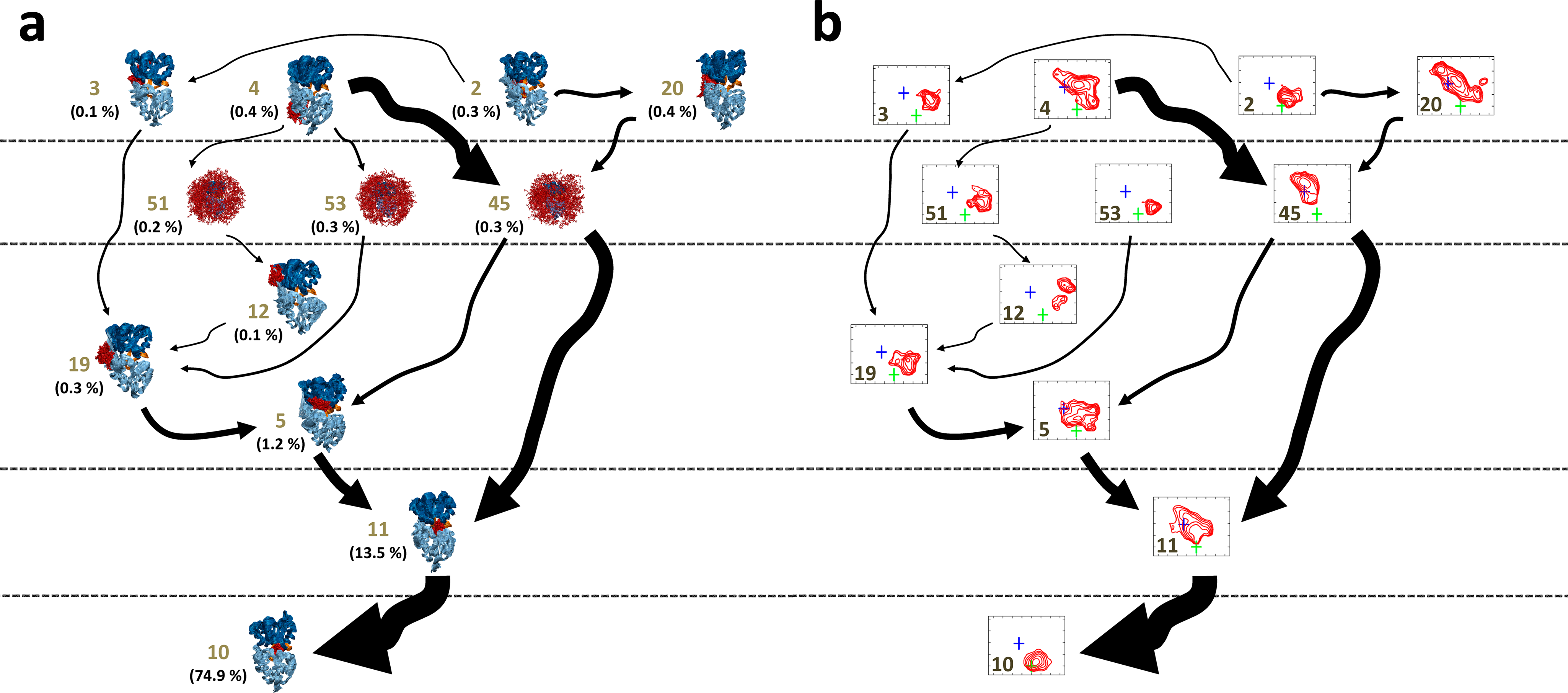

Supplement: Figure S5 — Superposition of the 5 highest flux pathways from mis-bound states to the bound state. The flux was calculated using a greedy backtracking algorithm from a 53-state Markov State Model (MSM) generated with the SHC algorithm. The arrow size is proportional to the interstate flux. (a) Representative structures and equilibrium populations are shown for each state. (b) Free energy plots of protein opening versus twisting angle are shown. The macrostate number is inserted in each of the free energy plots. (TIF) [file pcbi.1002054.s005.tif]

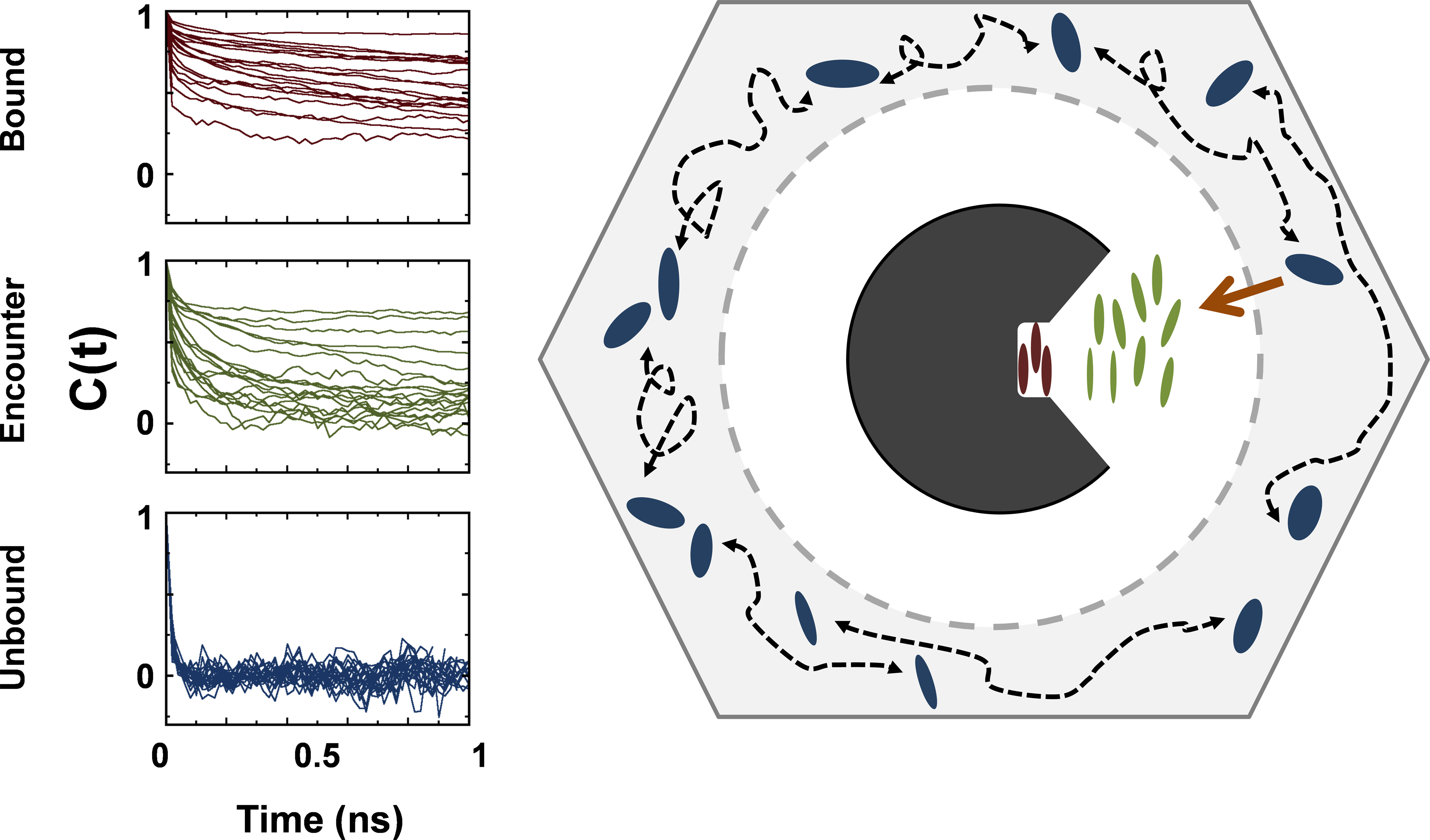

Supplement: Figure S6 — The ligand distance to the binding site vs. simulation time was plotted for 12 MD simulations where binding (11 simulations) or unbinding (1 simulation, gray shaded) events were observed. (TIF) [file pcbi.1002054.s006.tif]

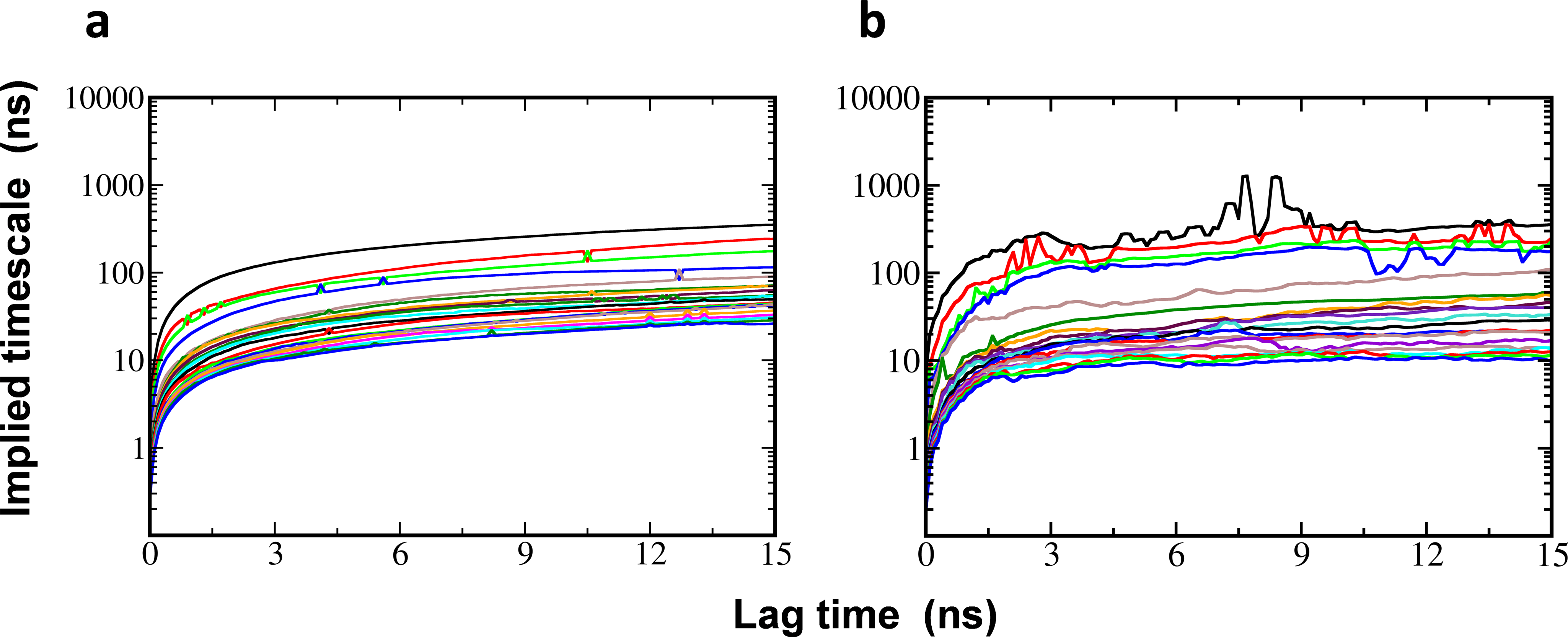

Supplement: Figure S7 — Ligand rotational autocorrelation functions for the unbound states (blue), the encounter complex state (green), and the bound state (red) are shown in the left panel. Autocorrelation functions generated from different trajectories are overlaid in the same figure. On the right panel, a schematic figure illustrates that the ligand rotates quickly when it is far away from the protein but the ligand rotation is restrained when it interacts with the protein. Thus, when constructing MSMs, we only consider the ligand center of mass motion when the ligand does not have strong interactions with the protein but we consider motion of all the ligand heavy atoms when the ligand is strongly interacting with the protein. (TIF) [file pcbi.1002054.s007.tif]

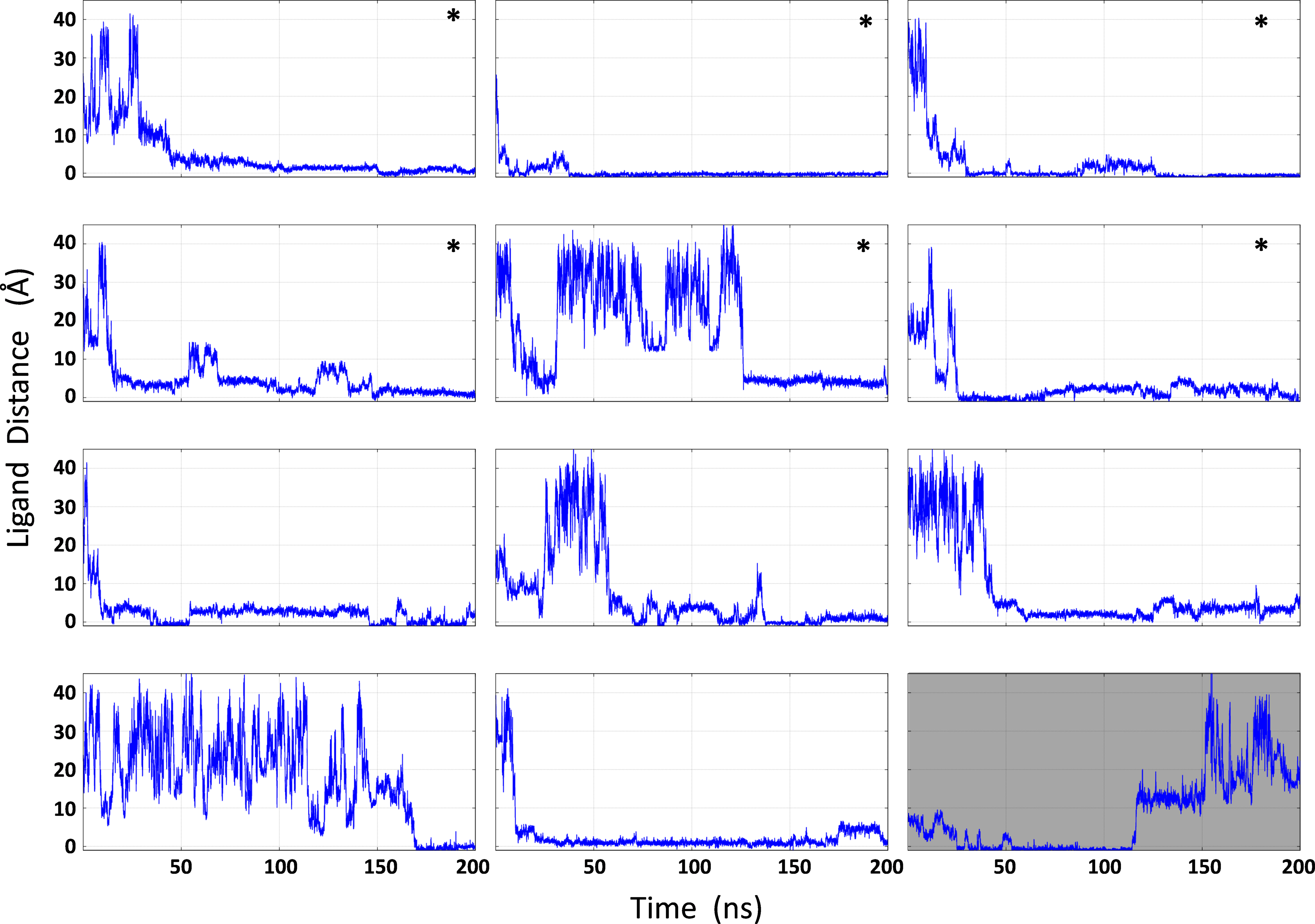

Supplement: Figure S8 — Twenty slowest implied timescales as a function of lag time computed from (a) MSM containing 3730 microstates and (b) MSM containing 54 macrostates. Both plots level off at a lag time of ∼4 ns. Thus we choose 6 ns to construct final MSMs. (TIF) [file pcbi.1002054.s008.tif]
