# Supplementary material for: A Role for Both Conformational Selection and Induced Fit in Ligand Binding by the LAO Protein
Source: PLoS Comput Biol. 2011 May 26;7(5):e1002054. doi: 10.1371/journal.pcbi.1002054 (PMC3102756; doi:10.1371/journal.pcbi.1002054)
Supplement: Table S1 — Averaged RMSD of the LAO protein Lobes I and II between three representative encounter complex conformations and the apo and bound X-ray structures. Cα atoms of the Lobe I residues 6-88 & 195-227 or Lobe II residues 92-185 were included in the RMSD calculations. The structural alignment and RMSD calculation were performed separately for each Lobe. (DOC) [file pcbi.1002054.s009.doc]

**Table S1**

| **Lobe I** | | **Lobe II** | |
| --- | --- | --- | --- |
| Apo (2LAO) | Closed (1LAF) | Apo (2LAO) | Closed (1LAF) |
| 1.61Å | 1.71Å | 1.94Å | 1.92Å |
