# Supplementary material for: A Role for Both Conformational Selection and Induced Fit in Ligand Binding by the LAO Protein
Source: PLoS Comput Biol. 2011 May 26;7(5):e1002054. doi: 10.1371/journal.pcbi.1002054 (PMC3102756; doi:10.1371/journal.pcbi.1002054)
Supplement: Text S1 — Structural features of the macrostates. (PDF) [file pcbi.1002054.s010.pdf]

Text S1: Structural features of the macrostates

Here we describe the features of each of the 23 most populated macrostates in our MSM (see Fig. S3 for representative conformations of the states):

- State 10 is the bound state. As discussed in the main text, it has an RMSD to the crystal structure of the binding site (PDB ID: 1LAF) as little as 1.2Å (see Fig. 2a). Moreover, the crystal structure of the bound state lies within the minimum of the most populated free energy basin (see Fig. 2). The average opening angle (10.5°), twisting angle (13.0°), and ligand distance (0.6Å) are also small, and thus close to the X-ray bound structure.

- State 11 is the major encounter complex (population 13.4%). In this state, the ligand is in or close to the binding site. Moreover, conformations in this state have small twisting (average=-1.2°), but large opening angles (average = 33.2°). State 5 (population 1.15%) also has features of the encounter-complex, though it plays a significantly more minor role. Protein conformations in state 5 are very similar to those in state 11, but the ligand is located further away from the binding site compared to State 11. As shown in Fig. 3, the majority of transitions out of state 5 go to state 11. However, there is a small probability for state 5 to directly transit to state 10 after one lag time (6ns). This transition is not among the 10 highest flux binding pathways, but does rank in the top 30 pathways.

- States 2, 4 and 9 are miss-bound states, where the ligand interacts with the protein outside and far from the binding site (see Fig. S3 for representative conformations from these states).

- States 1, 6, 7, 12, 13, 14, 18, 19, 20, 23 and 26 are intermediate states between unbound/misbound states and the encounter complex. In these states, the ligand is interacting with the protein at or near its binding site, but the protein is still quite open or twisted. Some of the intermediate states, like state 20 (See Fig. 3 and Fig. S5), are misbound states that can also play a role as intermediates in minor binding pathways.

- States 43, 45, 48, 51, 52 and 53 are the solvated states. Conformations in these states sample a wide range of opening and twisting angle, but share the common feature that the ligand is far from the active site (ligand distance is ~30 Å)
