# Supplementary material for: A Role for Both Conformational Selection and Induced Fit in Ligand Binding by the LAO Protein
Source: PLoS Comput Biol. 2011 May 26;7(5):e1002054. doi: 10.1371/journal.pcbi.1002054 (PMC3102756; doi:10.1371/journal.pcbi.1002054)
Supplement: Text S2 — Binding/unbinding transitions observed in the MD dataset. (PDF) [file pcbi.1002054.s011.pdf]

Text S2: Binding/unbinding transitions observed in the MD dataset

Among our 65 MD simulations, we have observed multiple binding events and unbinding events. In particular, 11 simulations started from the solvated (or unbound) states end in the encounter complex or bound states, and one simulation started from the bound state ends in the unbound state. Importantly, many of these simulations contain multiple transitions between the unbound, encounter complex, and bound sates (unbound to encounter complex: 903 transitions, and encounter complex to bound: 6529 transitions); all of which help us to obtain improved statistics. The transitions were counted using a sliding window of 6ns on sixty-five 200ns trajectories each contains 10,000 conformations (see “Calculating transition matrices” of the Methods section for more details). The presence of multiple transitions is strongly hinted at by a plot of the ligand distance versus the simulation time for the 12 simulations mentioned above (Fig. S6).
