# Supplementary material for: A Role for Both Conformational Selection and Induced Fit in Ligand Binding by the LAO Protein
Source: PLoS Comput Biol. 2011 May 26;7(5):e1002054. doi: 10.1371/journal.pcbi.1002054 (PMC3102756; doi:10.1371/journal.pcbi.1002054)
Supplement: Text S3 — Implied timescales calculation. (PDF) [file pcbi.1002054.s012.pdf]

Text S3: Implied timescales calculation

Examining the behaviors of the implied timescales is one way to check if the model is Markovian [1]. Implied timescales (τ_k) can be calculated as below:

$$\tau_k = -\frac{\tau}{\ln \mu_k(\tau)} \quad (1)$$

where μ_k is an eigenvalue of the transition matrix with the lag time τ. Each implied timescale describes an aggregate transition between subsets of macrostates. If the model is Markovian and Eq. (1) holds, the exponentiation of T should be identical to an MSM constructed with a longer lag time, and the implied timescales will be independent of the lag time.

The implied timescales of our model level off at a lag time of ~4 ns, indicating the model is Markovian (Fig. S8b). Thus, we chose a lag time of 6 ns. The dominant macrostates from our model are displayed in Fig. S3.

1. Swope WC, Pitera JW, & Suits F (2004) Describing Protein Folding Kinetics by Molecular Dynamics Simulations. 1. Theory. *J. Phys. Chem. B* 108:6571-6581.
